# Qualitative study of clinician and patient perspectives on the mode of anaesthesia for emergency surgery

**DOI:** 10.1002/bjs.11243

**Published:** 2019-08-01

**Authors:** J. Dooley, R. A. Armstrong, M. Jepson, Y. Squire, R. J. Hinchliffe, R. Mouton

**Affiliations:** ^1^ Department of Population Health Sciences Bristol Medical School Bristol UK; ^2^ Bristol Surgical Trials Centre Bristol Medical School Bristol UK; ^3^ Anaesthetic Department Southmead Hospital Bristol UK

## Abstract

**Background:**

Although delivering a chosen mode of anaesthesia for certain emergency surgery procedures is potentially beneficial to patients, it is a complex intervention to evaluate. This qualitative study explored clinician and patient perspectives about mode of anaesthesia for emergency surgery.

**Methods:**

Snowball sampling was used to recruit participants from eight National Health Service Trusts that cover the following three emergency surgery settings: ruptured abdominal aortic aneurysms, hip fractures and inguinal hernias. A qualitative researcher conducted interviews with clinicians and patients. Thematic analysis was applied to the interview transcripts.

**Results:**

Interviews were conducted with 21 anaesthetists, 21 surgeons, 14 operating theatre staff and 23 patients. There were two main themes. The first, impact of mode of anaesthesia in emergency surgery, had four subthemes assessing clinician and patient ideas about: context and the ‘best’ mode of anaesthesia; balance in choosing it over others; change and developments in anaesthesia; and the importance of mode of anaesthesia in emergency surgery. The second, tensions in decision‐making about mode of anaesthesia, comprised four subthemes: clinical autonomy and guidelines in anaesthesia; conforming to norms in mode of anaesthesia; the relationship between expertise, preference and patient involvement; and team dynamics in emergency surgery. The results highlight several interlinking factors affecting decision‐making, including expertise, preference, habit, practicalities, norms and policies.

**Conclusion:**

There is variation in practice in choosing the mode of anaesthesia for surgery, alongside debate as to whether anaesthetic autonomy is necessary or results in a lack of willingness to change.

## Graphical abstract

Although delivering a chosen mode of anaesthesia for certain emergency surgery procedures is potentially beneficial to patients, it is a complex intervention to evaluate. Seventy‐nine interviews with clinicians and patients were analysed thematically to explore experiences of anaesthesia in emergency surgery. The results highlight several interlinking factors affecting decision‐making, including expertise, preference, habit, practicalities, norms and policies.

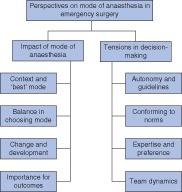

General anaesthesia or not?

## Abstract (es)

**Antecedentes:**

Si bien determinados tipos de anestesia en ciertos procedimientos quirúrgicos de urgencia pueden ser potencialmente beneficiosos para los pacientes, la decisión de su utilización es difícil de evaluar. Este estudio cualitativo exploró las perspectivas del clínico y del paciente sobre sobre el tipo de anestesia en cirugía de urgencia.

**Métodos:**

Se utilizó un muestreo de bola de nieve para reclutar participantes de 8 corporaciones del *National Health Service* (NHS) que cubrían tres entornos de cirugía de urgencia: rotura de aneurismas aórticos abdominales, fracturas de cadera y hernias inguinales. Un investigador cualitativo realizó 79 entrevistas a 21 anestesistas, 21 cirujanos, 14 técnicos de quirófano y 23 pacientes. Se realizó un análisis de las transcripciones de la entrevista por temas.

**Resultados:**

Dos fueron los temas principales. El primero era el impacto del tipo de anestesia en la cirugía de urgencia y tenía 4 subtemas que evaluaban las ideas del clínico y del paciente sobre: el contexto y el mejor tipo de anestesia, las ventajas e inconvenientes de la elección de un tipo sobre los otros, los cambios y avances en anestesia, y la importancia de la anestesia en cirugía de urgencias. El segundo era las dificultades en la toma de decisiones sobre el tipo de anestesia y comprendía 4 subtemas: la autonomía clínica y las guías de anestesia, el seguimiento de las normas en el tipo de anestesia, la relación entre experiencia, preferencia y opinión del paciente, así como las dinámicas del equipo en cirugía de urgencia. Los resultados resaltaron varios factores interconectados que afectan la toma de decisiones, incluyen la experiencia, las preferencias, los hábitos, algunos aspectos prácticos, las normas y las políticas.

**Conclusión:**

En la práctica, existen variaciones en la elección del tipo de anestesia para la cirugía, junto con el debate sobre si la autonomía anestésica es necesaria o si resulta en una falta de voluntad para el cambio.

## Introduction

It is possible to perform some surgical procedures under general anaesthesia (GA), neuroaxial regional anaesthesia (RA) or local anaesthesia (LA). Evidence is mixed as to the benefit of one mode of anaesthesia over another^1^, ^2^, ^3^. GA is the traditional method for providing anaesthesia and allows the safe, stable performance of surgical procedures^4^. There is, however, evidence that avoidance of GA may improve patient outcomes, especially in some emergency surgery settings^5^, ^6^. Multiple observational studies and a recent meta‐analysis of seven randomized trials have shown an association between GA and poorer outcomes after stroke thrombectomy^7^, ^8^. This growing evidence base has led to LA becoming standard care in many centres.

In patients who underwent emergency inguinal hernia repair, observational studies^9^, ^10^ indicated that LA carried a lower mortality risk, fewer cardiac and respiratory complications and faster recovery time. A *post hoc* subgroup analysis^11^ of a recent RCT comparing open and endovascular repair (EVAR) of ruptured abdominal aortic aneurysms (rAAAs) demonstrated a reduced 30‐day mortality rate for ruptured EVAR performed under LA compared with GA. A recent analysis^12^ confirmed that the use of LA for ruptured EVAR has been adopted in the UK with lower mortality rates with use of LA than GA. No prospective clinical trials have directly addressed the effect of mode of anaesthesia on outcomes in patients undergoing emergency vascular surgery. Similarly, the published evidence comparing different types of anaesthesia for hip fracture surgery is largely observational^13^, ^14^. As a consequence, professional guidelines for both hip fracture and aortic surgery are unable to recommend any particular anaesthetic technique over another.

Although delivering a particular method of anaesthesia for certain surgical procedures is potentially beneficial to patients, it is a complex intervention to evaluate through clinical trials. Qualitative research methods are integral to understanding practice, particularly in complex settings such as emergency surgery. This study aimed to address the fact that very little is known about clinician and patient preferences for the mode of anaesthesia and the rationale for selecting it in this setting.

## Methods

The National Health Service (NHS) Health Research Authority gave ethical approval for the study (REC reference 17/SC/0548).

Participants were recruited from three emergency surgery clinical contexts: rAAAs, hip fractures and inguinal hernias. These three procedures were chosen to represent different settings where GA, RA and LA are used routinely. Interviews were conducted between December 2017 and July 2018 with clinicians and patients from eight NHS Trusts. The semistructured interviews followed a topic guide to ensure that the same questions were asked to all participants, but allowed deviation in discussions according to the answers given. The sample size was not predetermined; instead, the aim was to maximize a range of views from different disciplines and settings within the data collection time frame and to conduct interviews until data saturation was reached^15^. Staff were recruited by snowball sampling: initial recruitment was done via e‐mail invitation to surgeons and anaesthetists with experience undertaking one (or more) of the three procedures, and participants then identified colleagues who could be recruited. Patients were sampled purposively from recent operating lists, and all had undergone emergency abdominal aortic aneurysm, inguinal hernia or hip fracture surgery between 3 months and 2 years before interview. Initial contact was made by telephone, which was followed by a letter and information sheet to those interested in participating. The anaesthesia technique was recorded from the clinical notes.

There were separate interview topic guides for clinicians and patients (*Appendix* S1, supporting information). The clinician topic guide was developed with guidance from two clinical authors to reflect current practice. The patient topic guide was developed with guidance from the Bristol vascular surgery patient focus group (meeting date 7 November 2017).

An experienced qualitative researcher conducted the interviews and analysed the transcripts using thematic analysis. Thematic analysis is an inductive method, meaning that analysts approach the transcript without a predefined theory and themes are generated through rigorous coding^16^. Each transcript was coded line‐by‐line according to content and meaning. Codes were then grouped to form categories first based on content. These were discussed and recategorized with all authors to identify themes and subthemes. Further analysis was conducted to develop the themes, which were modified at three further analysis meetings with all authors. Any variation according to clinician type or surgical setting was noted. The codes were applied to the transcripts and categorized using NVivo version 11 (QSR International, Burlington, Massachusetts, USA).

## Results

Interviews lasted between 18 and 60 min. Data saturation was reached after interviews with 23 patients and 56 clinician participants, including consultant anaesthetists (21), consultant surgeons (21) and other members of the emergency surgical team (14) from three specialty areas: fractured neck of femur (35), inguinal hernia repair (21) and EVAR for rAAA (21). Of the 23 patients interviewed, ten had surgery for fractured neck of femur, seven for strangulated inguinal hernia and six for rAAA (*Table* 1). Nineteen clinician and seven patient interviews took place in person; the remaining 37 clinician and 16 patient interviews were conducted by telephone.

**Table 1:** Clinician and patient characteristics

	*n*
**Clinician type**	
Anaesthetist	21
Surgeon	21
Nurse	9
Geriatrician	2
Interventional radiologist	2
Physician's assistant	1
**Clinician surgery type** *	
Hip fracture	35
Hernia	21
Emergency EVAR for rAAA	21
**Patient surgery type**	
Hip fracture	10
Hernia	7
Emergency EVAR for rAAA	6

* Some clinicians talked about more than one setting. EVAR, endovascular aneurysm repair; rAAA, ruptured abdominal aortic aneurysm.

**Figure 1:**
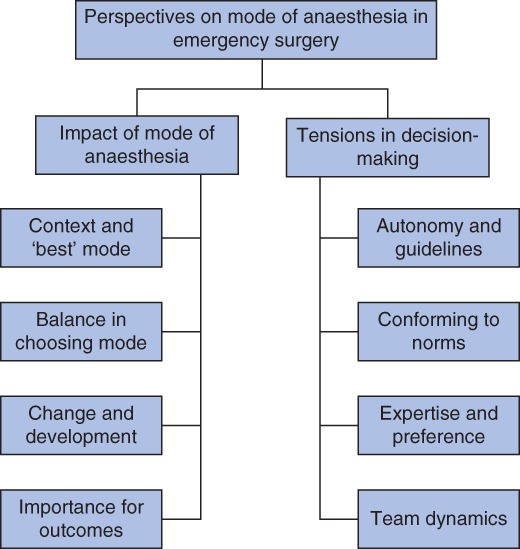
Thematic map of themes and subthemes as identified from interviews

Two themes and eight subthemes are reported alongside quotations from the interviews (*Table* 2) and a thematic map (*Fig*. 1).

**Table 2:** Themes and subthemes from thematic analysis with example quotations

Themes and subthemes	Example quotations
**Impact of mode of anaesthesia in emergency surgery**	
Context and ‘best’ mode of anaesthesia	There are a number of factors I take into consideration. There are patient factors. There are anaesthetist factors and team factors and surgeon factors (surgeon, vascular surgery, site 1) I'm assuming you're thinking that regional's going to be better for some patients? (anaesthetist, all settings, site 8) Presumably, it's thought of as beneficial because you're less affected by the anaesthesia when you have an epidural (patient having hip fracture surgery)
Balance in choosing mode of anaesthesia	They have their general anaesthesia, [the BP] plummets down to 40 or 50 [mmHg] and you have to get the cross‐clamp over quickly. If they're awake, they don't have that. But, equally, if they're thrashing around in agony then it's a very difficult thing to do (surgeon, hip fracture surgery, site 4) I had a choice of having an epidural or general and I went, ‘No, you put me to sleep.’ [Regional anaesthesia] would be ideal for me because a general anaesthetic doesn't agree with me. I just always make sure I've got plenty of [sick] bowls next to me (patient having hip fracture surgery)
Change and development in anaesthesia	Because we're better with our nerve blocks they are getting analgesia, whereas actually the best way to analgise them back then would have been to do a spinal (anaesthetist, hip fracture surgery, site 2) We've done a few more recently under local anaesthesia… there is emerging evidence that for ruptures, local anaesthesia gives a better outcome than general anaesthesia (interventional radiologist, EVAR, site 1) I didn't feel as bad this time with whatever they used as I have done before (patient having hernia repair)
Importance of mode of anaesthesia for outcomes in emergency surgery	It's always really hard to filter out what's the effect of the surgery and what's the effect of the anaesthetic (anaesthetist, site 2) No one sat down and explained to me what they were actually going to do – you know, with the anaesthetic. That would've helped me feel a lot easier (patient having hernia repair) If I'd have had local anaesthetic then I might not have been as groggy. I know when I woke up I was all over the place (patient having hernia repair)
**Tensions in decision‐making about mode of anaesthesia**	
Clinical autonomy and guidelines in anaesthesia	It's a blessing and a curse in terms of it's good that we take every patient and every situation on its merits and make a decision. But I think it does lead to excess variation in practice sometimes (anaesthetist, all settings, site 7) We've issued guidance and recommendations, and had anaesthetists try to talk to anaesthetists, and other anaesthetists try to cajole anaesthetists, and even just have a majority view, and we failed (surgeon, hip fracture surgery, site 6)
Conforming to norms in mode of anaesthesia	It is left to the discretion and the experience of the anaesthetist, but certainly the way things are done here it's more traditionally people are used to looking after people who have had a general anaesthetic (anaesthetist, all settings, site 2) [By using local anaesthesia] we managed to return [the patient] to a state where he was discharged from the hospital physiologically good about three or four days later. If he'd had an open triple‐A repair he would have been there for yonks, if he'd survived… [The surgeon] was very concerned even after having done the procedure that she was going to be hauled over the coals (anaesthetist, all settings, site 6)
Relationship between expertise, preference and patient involvement	Perhaps it's the luck of the draw – who you get. It's a bit like a doctor. You get good ones and you get bad ones (patient having hip fracture surgery) I personally do ask patients what they prefer, but I also inform their decision… I can't force them to do one thing or another, but equally I can tell them what my experience is and what other people would do (anaesthetist, hip fracture surgery, site 1) They wanted to do it while I was awake with a local. But I said to them, ‘If you do that, then I will start passing out and fainting’… So, they gave me something that made me really woozy and that was okay (patient having EVAR) They said, ‘Oh, it may be a spinal.’ I said, ‘Oh, yes, I'd rather have that,’ but then when I spoke to the anaesthetist he advised a general. I said to him, ‘I've got a terrible phobia of it,’ and he said, ‘Oh, I wouldn't worry about that too much’ (patient having hip fracture surgery)
Team dynamics in emergency surgery	So there's a whole team with a different ethos on different days (surgeon, site 1) Often, what the surgeon wants is different from the anaesthetist is willing to deliver (surgeon, hip fracture surgery, site 7)

EVAR, endovascular aneurysm repair.

### Theme 1: impact of the mode of anaesthesia in emergency surgery

#### *Context and ‘best’ mode of anaesthesia*

**Figure 2:**
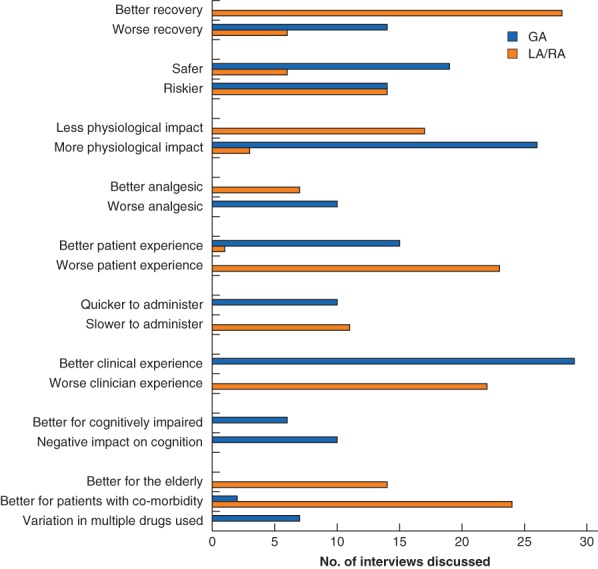
Advantages and disadvantages of general and local/regional anaesthesia GA, general anaesthesia; LA/RA, local/regional anaesthesia.

The potential advantages and disadvantages of GA, LA and RA were discussed (*Fig*. 2). LA and RA were described as having less physiological impact and hence being better for elderly patients or those with cardiovascular co‐morbidities, and for enabling a quicker recovery. GA was seen to provide a more comfortable experience for patients while making the technical side of surgery easier, as well as being quicker to administer and thus increasing theatre efficiency.

Both clinicians and patients assumed that the research team, by encouraging comparison of types of anaesthesia, were implying that LA or RA is preferable to GA. However, clinicians very rarely stated categorically that one form of anaesthesia was the best; most said it was context‐dependent. For example, some clinicians said GA was worse for influencing postoperative cognition, but it was also stated that it was better for those with existing dementia. This was primarily in hip fracture settings: patients with advanced dementia may lack cooperation for regional techniques, whereas GA prevented them becoming confused or agitated during the procedure. Additionally, GA and LA/RA were each reported as riskier than the other by equal numbers of people. Over half of the clinicians (31 of 56) stated explicitly that there was not a ‘best’ mode of anaesthesia. Over one‐third (20 of 56) cited previous research that had not shown differences in outcomes between modes of anaesthesia.

Fifteen of the 18 patients who stated a preference said they would prefer GA because of fear of awareness in surgery. Many of the patients had experience of LA or RA in elective operations, and this was seen as more acceptable than having LA or RA in emergency contexts. Patients reported that it was difficult to understand what surgery is like without experience; people who have not had LA or RA would be apprehensive about being awake in surgery, but experiences showed that it was not as bad as many people thought. However, almost half the patients (11 of 23) were unsure which form of anaesthesia they received, and in three instances the patient‐reported mode of anaesthesia differed from that recorded in the clinical notes.

#### *Balance in choosing mode of anaesthesia*

The decision about mode of anaesthesia was described as a balancing act between various competing factors, for example potential side‐effects *versus* the technical aspects of surgery, or patient experience *versus* physiological impact. There was a consensus that GA should be avoided when patients were more unwell. Where contraindications to particular anaesthesia techniques were discussed, there was debate about even those most commonly cited. For example, 18 clinicians cited a patient being on clopidogrel as a reason to avoid RA, whereas four said RA was still possible.

Patients also balanced different factors when expressing their preferences. For example, one patient still expressed a preference for GA over RA in spite of previous postoperative nausea and vomiting after GA.

#### *Change and development in anaesthesia*

Half of the clinicians (28 of 56) said their perspective on anaesthesia techniques had changed over time. Clinicians from hip fracture settings spoke about increasing their use of GA owing to national audits that showed little difference in outcomes, as well as the advent of nerve blocks to aid postoperative pain relief. Reported use of LA was increasing in ruptured EVAR settings, primarily due to research evidence.

Improvements in anaesthetic agents over time were seen to have decreased the physiological risks of GA. Patients who had undergone multiple anaesthetics over time also reported an improvement, primarily in how they felt in the immediate postoperative period.

#### *Importance of mode of anaesthesia for outcomes in emergency surgery*

There was disagreement about the importance of mode of anaesthesia in influencing patient outcomes after emergency surgery. Factors cited as being more impactful included care received after diagnosis, use of opiates, BP monitoring and duration of surgery. The most important factor was for the anaesthesia to be of high quality. This was described as keeping doses of anaesthetic drugs low, consistency within hospitals, and good communication with patients.

For patients, the most salient aspect of their recovery was the quality of care after surgery. However, many reported vivid memories of their experience of the anaesthetic, as well as highlighting the importance of communication in preparing them for the anaesthetic experience.

### Theme 2: tensions in decision‐making about mode of anaesthesia

Many competing factors affect clinicians' decision‐making about anaesthesia (*Table* 3), causing tensions.

**Table 3:** Reported factors that affect clinicians' decision‐making about mode of anaesthesia

	No. of interviews (*n* = 56)
Practical factors	34 (61)
Norms and habits	22 (39)
Surgical factors	16 (29)
Time	16 (29)
Clinician expertise	44 (79)
Concern about converting to general anaesthesia	13 (23)
Clinician preference	34 (61)
Patient preference	36 (64)

Values in parentheses are percentages.

#### *Clinical autonomy and guidelines in anaesthesia*

Anaesthetists described a clinical autonomy that was central to their training and practice, reporting regular monitoring of their practice and use of current research to inform decision‐making. However, 16 clinicians reported that, although this allowed decision‐making to the benefit of individual patients, it could lead to a concerning variation in practice.

Most sites reported not having guidelines for anaesthesia. Some hospitals described ‘recipes’ being in place that represented guidance rather than being prescriptive. Clinicians in hip fracture settings most commonly reported the use of local guidelines, often as a result of findings from the National Hip Fracture Database. However, autonomy was still seen as a priority in anaesthesia, which resulted in resistance to policy in some Trusts.

#### *Conforming to norms in mode of anaesthesia*

Alongside the description of autonomy there was much, potentially contradictory, discussion of conforming to norms – default modes of anaesthesia within teams. In hip fracture settings, clinicians described norms within hospitals but variation between hospitals, echoing the findings from the National Hip Fracture Database. In ruptured EVAR settings, GA was most prevalent, with all but one hospital site reporting almost always using GA for ruptured aneurysms. GA was seen as a national norm for emergency hernia repairs. Where clinicians reported LA, this was a personal deviation based on specific expertise.

Clinicians reported a tension between autonomy and norms, with a sense that the default technique used in a certain hospital takes priority over clinical judgement. There was a reported tendency in surgery to stick with established methods. These habits became ingrained in hospital culture, resulting in feeling exposed if one tried to break the norm.

Practical factors also contributed to GA being a default method. Clinicians from four sites reported difficulty in accessing the necessary theatres or staff to undertake procedures under LA, resulting in GA being the only option for abdominal aortic aneurysm repairs. In hip fracture settings, a lack of consistency in staffing trauma lists resulted in non‐specialists anaesthetizing many patients, leading to greater use of GA. Clinicians in hernia settings cited the clinical uncertainty of a patient needing emergency hernia repair as a reason to use GA; the possibility of bowel involvement meant that LA or RA could never be used.

#### *Relationship between expertise, preference and patient involvement*

Forty‐four clinicians said that expertise was a key component of anaesthesia choice. Most spoke about this in general terms, but ten said that it was down to anaesthetist skill and six specifically to surgeon skill. GA was seen to be the type of anaesthesia that people were most comfortable with. In EVAR and hernia settings, variation in training and a lack of experience doing these procedures under LA was seen as a reason to choose GA.

Expertise led to preferences that then guided decisions about anaesthesia, which was seen as the cause of variation in practice. Variation was also recognized by patients. Over half of clinicians (36 of 56) reported that patient preference was taken into account, but half of these described guiding patients towards a particular choice.

Half of the patients reported that they did not have, or could not remember having, a discussion about the mode of anaesthesia. The rest described different levels of involvement; some felt their opinions were considered, whereas others felt the opposite.

#### *Team dynamics in emergency surgery*

A positive team dynamic was seen as very important for quality decision‐making. However, this was reported as challenging in emergency surgery where the ever‐changing staff lists meant that relationships were harder to form than in elective settings.

Nearly three‐quarters of clinicians said the anaesthetist was ultimately responsible for decisions about the method of anaesthesia. This was reported equally by surgeons and anaesthetists, and nearly all other theatre staff. Over half of clinicians reported surgeon involvement in decision‐making about the mode of anaesthesia. This varied according to setting, with surgeons reported as most involved in EVAR (37 reporting surgeon involvement) and least involved in hip fracture settings (24 reporting surgeon involvement).

Anaesthetists and surgeons each spoke about how the other affects the choice of mode of anaesthesia. Half the anaesthetists (11 of 21) made references to surgeons misunderstanding aspects of anaesthesia, which in some instances resulted in a preference for GA. Eight surgeons described variations in opinions and preferences between anaesthetists for the same surgical procedure, resulting in a different mode of anaesthesia depending on the individual present for the procedure.

## Discussion

The mode of anaesthesia used in emergency surgery can depend on habit, expertise, preference, practicalities and organizational norms or policies, all of which are interlinked. The themes identified in this analysis highlight the tension between clinician and patient preferences and beliefs about the different modes of anaesthesia, and the practical or cultural factors that affect their use in practice.

The recognized variation in anaesthetic practice is attributed primarily to the need for clinician autonomy. To many clinicians, autonomy is key to high‐quality anaesthetic practice, and is emphasized in anaesthetic training. This reflects a scepticism of standardization in healthcare, which questions its utility and potential for curtailing professional judgement^17^. However, one of the most cited reasons for using one technique over another was that it was the norm or default within that organization. When discussing the reasons for norms developing, clinicians cited habits, expertise and preferences rather than research evidence or local or national guidelines. These local defaults were considered difficult to challenge, indicating that culture within a team or hospital site may take preference over autonomous judgement. In previous work, anaesthetists have defined their role as ‘a cog in the wheel’, giving their service to ensure that surgeon and patient experience is as smooth as possible^18^. This may explain why autonomy may be curtailed in place of trust norms: default modes of anaesthesia within teams. The tension between professional judgement and implementation of care pathways in clinical trials in emergency surgery has been described^19^. Clinicians were concerned about being able to follow pathways given the unpredictability of emergency surgery. However, rather than resistance or refusal, this led to flexibility in how they were implemented in different sites. The authors described this as clinician willingness to change practice in order to enhance quality of care.

The contribution of mode of anaesthesia to patient outcomes after emergency surgery was debated. There was disagreement over whether the short‐term effects of having GA compared with LA/RA ultimately affect patient recovery, given the multitude of other contributory factors. The presumption that LA/RA was better for patient outcomes was thus questioned. Although clinicians tended to focus on clinical outcomes such as mortality or duration of hospital stay, patients described positive or negative experiences in different terms. Many clearly identified anaesthesia as the causal factor in their experience of the perioperative period – whether it was feeling better after GA than they had following a previous operation, or vivid memories of how the anaesthetic made them feel on induction and emergence. This is also evident in the literature, with patients reporting anaesthetic‐related factors such as nausea or pain from the tracheal tube as important in their experience of surgery^20^.

This apparent disconnect between the outcomes clinicians used to inform their decision‐making and those patients felt were important needs to be considered when designing prospective trials comparing modes of anaesthesia. Ongoing efforts to standardize the endpoints reported in clinical trials will include patient‐centred outcome measures^21^, ^22^. Both patient‐reported outcome and experience measures are being used increasingly^23^. For example, a core outcome set has been developed for hip fracture trials, which identifies quality of life, pain and activities of daily living as three of the five core outcomes^24^. The extent to which these factors influence clinician decision‐making when selecting mode of anaesthesia is unclear. Although 64 per cent of clinicians reported patient preference as a consideration, half of the patients reported that they did not discuss choice of anaesthesia with an anaesthetist. Some patients felt that their experience would have been improved had they been better informed before anaesthesia. A minority of patients reported undergoing a form of anaesthesia that differed from that in the clinical record.

The existence of strongly held, contradictory opinions on modes of anaesthesia raises the question of whether autonomy leads to a dogma that prospective research could be used to address. Similar numbers of clinicians provided strong, convincing arguments for the benefits of both GA and RA for hip fracture surgery. Although fewer clinicians preferred LA for inguinal hernia repairs or EVARs, there were passionate advocates who were convinced of its benefit for patients. Many people cited research as a reason for holding either viewpoint, but this research provided evidence in support of contradictory opinions. Much of the published evidence comparing modes of anaesthesia comprises observational studies using large registries or databases such as the National Hip Fracture Database or National Vascular Registry. Although these studies include large numbers of patients, they are unable to control for variables such as the use of sedation or variability in drugs used. Similarly, an examination of existing Cochrane reviews on mode of anaesthesia found large variability within and between studies, which reduces the reliability of any conclusions drawn^25^. It is thus unsurprising that most clinicians provided nuanced reasoning based on team dynamics, expertise and experience, rather than research or evidence‐based decision‐making.

These findings should be considered in the context of the strengths and limitations of the study. Interviews were undertaken in eight NHS hospitals in the West of England, including large tertiary and smaller district general hospitals, illustrating a wide variety of practice. Seventy‐nine is a large sample size for a qualitative study and represents a range of perspectives. The inclusion of three different emergency surgery settings and different clinical specialties increases the generalizability of the results. Including the patient viewpoint allows a more inclusive clinical discussion. A limitation of this study is that the findings might not extrapolate to other geographical areas. Another potential limitation comes from the recruitment strategy, which resulted in a bigger representation of clinicians from hip fracture settings and fewer non‐surgeon/non‐anaesthetists in the final sample. Additionally, patients had experienced surgery up to 2 years before interview, which may have affected their memory of the operation.

The nature of anaesthesia will always necessitate flexibility in practice, based on clinical expertise, surgical need and patient factors. However, clinicians are aware of the unmonitored variation in practice, as well as practical and cultural constraints to decision‐making. High‐quality research, epitomized by RCTs, has been extremely important in changing practice in contexts where it had previously been thought impossible or unnecessary. This qualitative study has demonstrated that both clinicians and patients are amenable to further prospective research comparing modes of anaesthesia in emergency surgery in order to provide guidance to both groups in the process of shared decision‐making.

## Supporting information


**Appendix S1.** Supporting Information.
