# Supplementary material for: Qualitative study of clinician and patient perspectives on the mode of anaesthesia for emergency surgery
Source: Br J Surg. 2019 Aug 1;107(2):e142–50. doi: 10.1002/bjs.11243 (PMC6973173; doi:10.1002/bjs.11243)
Supplement: Supplementary file 1 — Appendix S1. Supporting Information. [file BJS-107-e142-s001.docx]

**BJS11243**

**Qualitative study of clinician and patient perspectives on the mode of anaesthesia for emergency surgery**

J. Dooley, R. A. Armstrong, M. Jepson, Y. Squire, R. J. Hinchliffe and R. Mouton

**Appendix S1 Interview Topic Guides**

**Clinician Topic Guide**

The aim of these interviews is to inform the design of a randomised controlled trial comparing the use of local, regional, and general anaesthesia in different emergency surgical contexts. For these interviews we are talking to people who are involved with non-elective EVAR (endovascular aneurysm repair), hip fracture, and hernia operations. These have been chosen because they are all common non-elective operations where at least two different anaesthesia types can be used. We want to find out the thoughts and preferences of clinicians in using different anaesthesia types.

- Check have received information sheet and consent form.
- (If on the phone) Ask for verbal consent to audio record, and ask to email/post signed consent form.
- (if face-to-face) Ask to sign consent form.
- Explain might use quotes in publications but will always be anonymous.

Press record – state the person has consented at the start of the recording.

**Background:**

- Can you describe your current position and role?
  - What sort of surgical procedures are you involved with?
  - How often are you involved in this type of surgery?
  - Would you say you were a specialist in a type of surgery or that you work more generally across different specialties?
    - If specialist, what type?
- How long have you been working on these types of surgery?

**LA, RA, GA:**

- What type of anaesthesia do you use when undertaking emergency EVAR/hernia/hip fracture surgery?
  - Why?
- Which type of anaesthesia do you think is better for EVAR/hernia/hip fracture surgery?
  - Why
- What other health professionals tend to be present when you work on these procedures?
- When you are in theatre, who has the ultimate responsibility for making decisions about the type of anaesthesia used?
  - How are these decisions made?
- In your experience, does the type of anaesthesia used for these procedures vary according to Hospital Trust/people you’re working with?
- What would affect which type of anaesthesia that is used?
  - Other clinicians/theatre staff present have a preference?
  - Patients have a preference?
  - Patient medical conditions?
  - Time/Personnel available
  - Trust or Dept policy, guidance documents, research papers
    - For all above, if so tell me more?
- I understand that while surgery can start off with LA or RA, clinicians can increase levels of sedation or anaesthetic as the surgery progresses.
  We are interested in how clinicians working in emergency surgery would define LA or RA versus GA. What are your thoughts?
  - Is this something you had considered previously?

**Closing**

- Is there something else you would like to add about what we have talked about today?
- Would you like to have a certificate for participating in this research?
- Do you know of any people you work with who you think might be willing to participate in an interview?
  - surgeons/anaesthetists/interventional radiologists/theatre staff

**Patient Topic Guide**

We are planning a research project comparing different types of anaesthesia for AAA/hernia/hip fracture surgery. We wanted to talk to you today about your experiences with surgery and your opinions on our planned project. This will help us design the research project from the perspective of patients.

- Obtain consent to audio record.
- Explain might use quotes in publications but will always be anonymous.

**Background**

- What surgery did you have?
  - How long ago?
- What was your experience of the surgery?
  - Experience leading up to emergency surgery – did you know anything was wrong?
  - Can you remember anything about the day the surgery happened?
    - Did any doctor explain the procedure before you went in for surgery?
  - How would you describe your recovery after your operation?
    - In hospital (complications, pain, emotional function)
    - At home (complications, pain, emotional function)
    - How long did it take?

**LA, RA, and GA**

- Do you know/remember what type of anaesthesia you had when you had your operation?
- Do you remember if any of the doctors explained the anaesthesia before you had your operation?
- What’s your understanding about the difference between LA, RA, and GA?
  - Explain the difference if not known.
- What’s your experience of LA, RA, and GA?

**Closing**

- Do you have anything else you would like to say about the planned research project?
- Is there anything else you would like to add about your surgery or any of the other things we have talked about today?
